# Editorial: Knowledge, attitude and practices of the public and healthcare-professionals towards sustainable use of antimicrobials: the intersection of pharmacology and social medicine

**DOI:** 10.3389/frabi.2024.1374463

**Published:** 2024-02-21

**Authors:** Márió Gajdács, Shazia Jamshed

**Affiliations:** ^1^ Department of Oral Biology and Experimental Dental Research, Faculty of Dentistry, University of Szeged, Szeged, Hungary; ^2^ Department of Pharmacy Practice, School of Pharmacy, International Medical University, Kuala Lumpur, Malaysia

**Keywords:** antibiotics, antimicrobial resistance, stewardship, sustainability, multidrug resistance (MDR), knowledge–attitudes–practices (KAPs)

Since their clinical introduction in the 1950s, antibiotics (ABs) have emerged as one of the most important groups of medicines. However, with the continuous emergence of multidrug-resistant (MDR) pathogens, antimicrobial resistance (AMR) threatens the treatment of life-threatening infections, the functionality of healthcare systems worldwide, and the attainment of the United Nations Sustainable Development Goals (UN SDGs) (Jasovsky et al., 2016; Gajdács et al., 2021). The two main hallmarks of AMR are the lack of novel antimicrobial agents and the imprudent use of existing drugs; this includes their prescription by relevant healthcare professionals in inappropriate indications (overuse) or doses (misuse) and their consumption by patients as a form of self-medication (Ahmed et al., 2023). ABs are often termed “social medicines,” as their misuse has wide-ranging global consequences, which is atypical of any other group of pharmaceuticals (Böhm et al., 2022). While the literature on the preclinical research and development of novel antimicrobials is extensive, the topic of societal, macro-economic, and behavioral aspects influencing AMR have been, up until recently, largely neglected, even though it has been shown that the knowledge and attitudes toward antimicrobials considerably influence practices associated with these drugs, both among professionals and laypeople (Cambaco et al., 2020; Maugeri et al., 2023). Thus, by devising the Research Topic “*Knowledge, attitude and practices of the public and healthcare-professionals towards sustainable use of antimicrobials: the intersection of pharmacology and social medicine*,” we aimed to enrich the literature pertaining to the perceptions of the public and the knowledge–attitudes–practices (KAPs) of healthcare professionals toward the use of antimicrobials and AMR, in addition to the relevance and efficacy of antimicrobial stewardship (AMS) in community and inpatient healthcare setting. Our aim was to aid future interventions and educational campaigns to increase awareness toward the importance of ABs and to reduce their inappropriate use. To our delight, four thematic articles, corresponding to a combined global authorship of 25 scientists, were published in our Research Topic, emphasizing the importance of government commitment, educational interventions, and public awareness campaigns to promote AMS, in addition to its non-negligible “One Health” aspect.

Nowadays, the development of clinical guidelines and recommendations depends on the continuous screening and evaluation of available evidence, to ensure only the most reliable scientific findings are being taken up into everyday practice. The systematic review and meta-analysis published by Gong et al. aimed to assess the efficacy and safety of the total glucoside of peony (TGP) extracted from a Chinese peony (“Bai shao”; *Paeonia lactiflora* Pall.), a medicinal herb used in traditional medicine, in combination with other conventional immunomodulators for the treatment of systemic lupus erythematosus (SLE). The study involved randomized controlled studies from eight scientific databases up to March 2022. Overall, a total of 23 articles that involved data for 792 patients in the treatment group and 781 patients in the control group, were assessed. A combination of TGP with conventional treatments (i.e., glucocorticoids, cyclophosphamide, and hydroxychloroquine) was deemed superior in controlling SLE disease activity and the incidence of adverse drug reactions when compared to conventional treatments alone. Furthermore, according to the published studies, TGP use had led to improvements in various SLE-related outcomes, such as recurrence rate and levels of complement protein and immunoglobulin. Overall, the study highlighted that TGP maybe a safe and effective adjunctive treatment for SLE; however, it also noted the poor quality of currently available evidence; thus, to ensure adequate rigor in clinical practice, randomized controlled trials (RCTs) with robust methodology are needed.

Injudicious use of ABs is one of the main hallmarks of AMR, especially in low- and middle-income countries, where laws and health-policy regulations implicitly allow for the selling of these drugs without prescriptions (Jacobs et al., 2019). The descriptive, cross-sectional survey study of Shitindi et al. aimed to compare the KAPs related to self-medication with antibiotics between medical (MED; n=400) and non-medical (non-MED; n=429) students at two MED and two non-MED universities in Dar es Salaam, Tanzania. The findings of their research showed that knowledge related to antimicrobials (as expressed by a knowledge score) was significantly higher among MED students. In addition, the easy access to a pharmacy, the over-the-counter availability of antibiotics, and lack of knowledge related to the risks of self-medication were critical factors facilitating non-prescription use. MED students were more likely to self-medicate; in addition, significant associations were identified between increasing age, attitude scores, and higher probability to self-medicate. On the other hand, sex, marital status, and year of study in their university program were not relevant predictors of self-medication. Even though their study targeted individuals attending higher education institutions, self-medication rates were still considerably high; thus, emphasizing the need for educational interventions and public awareness campaigns.

Mortality associated with AMR disproportionally affects various regions of the globe, with Africa bearing the greatest burden of mortality from drug-resistant infections (Antimicrobial Resistance Collaborators, 2022). This occurs in parallel with dramatic increases in global AB consumption, with low- and middle-income countries as the main contributors (Laxminarayan et al., 2013). Although initiatives targeting healthcare professionals are essential, without wide-ranging societal changes and commitment (both technical and financial) from national governments, notable changes in drug utilization practices may not be expected (Akpana et al., 2020). The study of Fuller et al. describes the progress of World Health Organization African region (WHO AFRO) member states in the implementation of strategies to optimize antimicrobial use (AMU). Out of the 47 countries, 31 participated; the WHO AMS assessment tool was used to survey relevant national policymakers on AMR, regarding the implementation of the core elements of AMS in their country. Overall, the findings of the study show that wide gaps exist in the implementation of AMS programs: only 25.8% of countries developed a national AMS implementation policy (with goals, targets, and operational plans), while the incorporation of the WHO Access, Watch, Reserve (AWaRe) classification and AMS practices into clinical guidelines was present in only 38.7% and 34.5% of countries, respectively. Furthermore, while regulations on non-prescription dispensing of ABs were present in 68% of countries, the enforcement of these laws is substandard. The study highlights the need for more commitment from government leadership to ensure the continent-wide sustainability of healthcare systems and the responsible use of antimicrobials.

The need for interdisciplinary, multisectoral, and collaborative efforts to tackle the issue of AMR has been front-and-center in recent decades (Olczak-Pienowska and Hryniewicz, 2021). This has been highlighted further by the “One Health” aspect of AMR, appreciating the interconnected nature of AMR with human health, animal and plant health, and the environmental aspects (e.g., pollution) (Kasanga et al., 2023). Redman-White et al. provided a comprehensive review on the predictors of farm-level AMU and AMR in food-production animals (focusing on pigs, layer and broiler hens, beef and dairy cattle, sheep, turkeys, and farmed salmon). Their article used a semi-systematic snowball approach, and included 179 peer-reviewed studies and 16 gray literature publications in English, from the Web of Science, SCOPUS, and MEDLINE databases up to May 2022. The review detailed the factors affecting AMU in each food-production animal group, the farming characteristics, the key stages of food production, the biosecurity risks, and then highlighted the interface between human and animal health in the context of AMR. Their work also highlighted that farm-level AMU analyses should focus on low- and middle-income countries, where livestock production is most rapidly expanding.

Antimicrobial drugs gained prominence not only because they can eradicate or minimize infections, but also because their overuse and misuse have emerged as major culprits of antimicrobial resistance, thus, also jeopardizing their efficacy potential. As antimicrobials are largely exploited for both human and veterinary consumption for prophylactic and therapeutic measures, extensive antimicrobial stewardship at the interdisciplinary and multisectoral levels should be the cornerstone of each developing and transitional economy.

## Author contributions

MG: Conceptualization, Writing – original draft, Writing – review & editing. SJ: Conceptualization, Writing – original draft, Writing – review & editing.
